# Natural Oregano Essential Oil May Replace Antibiotics in Lamb Diets: Effects on Meat Quality

**DOI:** 10.3390/antibiotics9050248

**Published:** 2020-05-12

**Authors:** Ivan A. Garcia-Galicia, Jose A. Arras-Acosta, Mariana Huerta-Jimenez, Ana L. Rentería-Monterrubio, Jose L. Loya-Olguin, Luis M. Carrillo-Lopez, Juan M. Tirado-Gallegos, Alma D. Alarcon-Rojo

**Affiliations:** 1 Facultad de Zootecnia y Ecología, Universidad Autónoma de Chihuahua, Chihuahua 31453, Mexico; igarciag@uach.mx (I.A.G.-G.); ing.jarras@gmail.com (J.A.A.-A.); arenteria@uach.mx (A.L.R.-M.); jtirado@uach.mx (J.M.T.-G.); 2 Catedrático CONACYT-UACH, Facultad de Zootecnia y Ecología, Universidad Autónoma de Chihuahua, Chihuahua 31453, Mexico; mhuertaj@uach.mx (M.H.-J.); lmcarrillo@uach.mx (L.M.C.-L.); 3 Posgrado en Ciencias Biológico Agropecuarias/Unidad Académica de Medicina Veterinaria y Zootecnia, Universidad Autónoma de Nayarit, Compostela 63700, Mexico; joselenin28@hotmail.com

**Keywords:** lamb, carvacrol, monensin, meat tenderness, TBARS

## Abstract

A study was conducted to investigate the effect of oregano essential oil (OEO) and monensin sodium on the oxidative stability, colour, texture, and the fatty acid profile of lamb meat (m. *Longissimus lumborum*). Twenty Dorper x Pelibuey lambs were randomly divided into five treatments; control (CON), monensin sodium (SM, Rumensin 200^®^ 33 mg/kg), a low level of OEO (LO, 0.2 g/kg dry matter (DM)), a medium level of OEO (MO, 0.3g/ kg DM), and a high level of OEO (HO, 0.4 g/kg DM). Dietary supplementation of OEO at any concentration lowered the compression strength in comparison with CON and SM. MO had the highest a* values (7.99) and fatty acid concentration (C16:1n7, C18:1n9c, C18:1n6c, C20:1n9, and C18:2n6c) during storage for 7 d at 3 °C. Lipid oxidation was not promoted (*p* > 0.05) by the moderated supplementation of oregano essential oil; however, OEO at 0.3 g/kg DM showed a slight lipid pro-oxidant effect. Dietary supplementation of MO and SM had the same effect on colour, tenderness, and the fatty acid profile of lamb (*L. lumborum*). It was demonstrated that oregano essential oil was beneficial for lambs feeding, and it could be a natural alternative to replace monensin in lamb diets with improvements in the quality of the meat.

## 1. Introduction

Feeding systems in animal production are using additives that improve growth. These additives modify the weight daily gain, composition and yield of carcass, nutritional value, stability, and shelf life of the meat. Nowadays, the consumers are demanding naturally raised, trusted, and organic meat and meat products. Hence, there is a need to look for alternatives to the use of synthetic growth promoters [1,2].

Monensin sodium (SM) is an antimicrobial ionophore produced by *Streptomyces cinamonensis* and is used in ruminants to improve feed efficiency [3]. Even though antimicrobial growth promoters in animals intended for human consumption are strongly limited in some countries [4,5], they are still used in the U.S.A. and Mexico.

Phytochemicals have properties that benefit not only the animals fed with them but indirectly might impact the consumers of the meat [6,7]. Oregano essential oil (OEO) has antioxidant and antimicrobial properties, due to its phenolic components, mainly thymol and carvacrol [8,9,10]. The active components (carvacrol and thymol) of OEO are potent antimicrobials affecting populations such as *E. coli*, *Staphylococcus aureus*, *Salmonella typhimurium*, protozoa, fungi, *Ruminococcus fibrisolvens,* and *Fibrobacter succinogenes*; this change in populations modifies ruminal fermentation, which is fundamental in the conversion of dietary nutrients to muscle tissue [11,12,13]. Specifically in sheep, there is evidence that carvacrol potentially decreases acetate concentrations, and it increases propionate and butyrate. Both are volatile fatty acids precursors of muscle and fat components in the animal [14].

Specific nutritional components in the lamb diet might directly affect the quality of the meat. Ruminant meat research has drawn considerable attention because ruminant meat contains some bioactive lipids, including n-3 long chain polyunsaturated fatty acids (n-3 LC-PUFA), and the fatty acid profiles of ruminant meat can be enhanced through dietary supplementation [15,16]. Polyunsaturated fatty acids (PUFAs) are not only essential nutrients for humans, but also significant in providing protection from cardiovascular diseases, inflammatory diseases, diabetes, some cancers and behavioral disorders [17,18,19]. Therefore, the consumption of adequate n-3 LC-PUFAs is crucial to maintaining a healthy body and for the prevention of chronic diseases [20]. Humans can obtain n-3 LC-PUFA or their C18 PUFA precursors from various sources including aquatic, farm livestock products, oilseeds, fruits, herbs, cyanobacteria, and microorganisms [21].

The formation of large amounts of saturated fatty acids (SFAs) in the rumen is a result of the biohydrogenation process when bacteria convert unsaturated fatty acids to SFA. So, the fatty acids (FAs) occurring in the rumen are highly saturated and take part in the absorption as well as deposition of the fat in muscles. The decrease in SFA and increase of health-beneficial fatty acids (PUFAs) content have been an important objective in ruminant meat studies. According to the Food and Agriculture Organization of the United Nations [22], the ratio of PUFA and SFA is a significant indicator for the nutritional evaluation of fat, with a recommendation of about 0.40. It is essential to increase the consumption of eicosapentaenoic (EPA) and docosahexaenoic acid (DHA) in human diets, because the synthesis of these FAs from dietary α-linolenic acid (ALA, C18:3 n-3) is very restricted [23].

Dietary supplementation of OEO in pigs, chickens, and cattle rendered meat with lower microbial concentration and higher antioxidant capacity [24,25,26,27,28,29,30]. Essential oils have shown to decrease methane production [31] and biohydrogenation [32]. The FAs profile in meat may be affected by the form of the provided lipid. However, the exact influence of oregano essential oil on the FA profile of lamb meat is limited.

Limited information exists on the benefits of phytochemicals in the productive performance and carcass and meat quality of ovine and other small ruminants. Moreover, information is available in terms of OEO and oxidative stability, but little is known about the effects on the fatty acid profile of lamb meat. Here, the aim of this study was to investigate the effect of the dietary supplementation of three levels of oregano essential oil and sodium monensin on the oxidative stability, colour, texture, and the fatty acid profile of lamb meat (m. *Longissimus lumborum*).

## 2. Results

### 2.1. Lipid Oxidation

**Figure 1:**
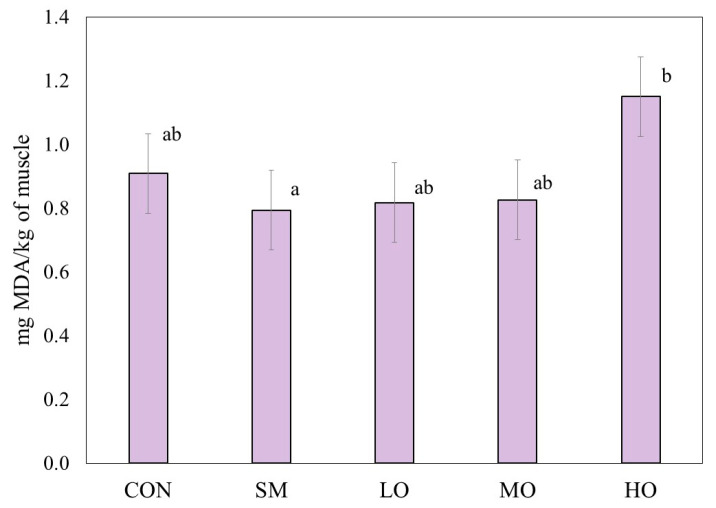
Means (± S.E.) of thiobarbituric acid reactive substances (TBARS) values (mg malonaldehyde (MDA)/kg of muscle) in m. *Longissimus lumborum* of male lambs unsupplemented (CON) or supplemented with monensin (SM), or three different levels of oregano essential oils: low (LO = 0.2 g/kg of dry matter (DM)); medium (MO = 0.3 g/kg of DM) or high (HO = 0.4 g/kg of DM). TBARS analysis was performed after 7 d of simulated retail display in modified atmosphere packaging (O_2:_CO_2_, 75:25%). ^a, b^ Different superscripts mean significant difference (*p* ˂ 0.05) among treatments within the variables.

Oregano essential oil (OEO) and monensin (SM) did not have advantages (*p* = 0.28) on the lipid oxidative stability of lamb (Figure 1). However, meat from male lambs fed with a high level of OEO (HO) had a 46% higher (*p* < 0.05) malonaldehyde (MDA) formation (1.15 ± 1.25 mg MDA/kg) when compared to SM (0.79 ± 1.25 mg MDA/kg).

### 2.2. Compression Strength

**Figure 2:**
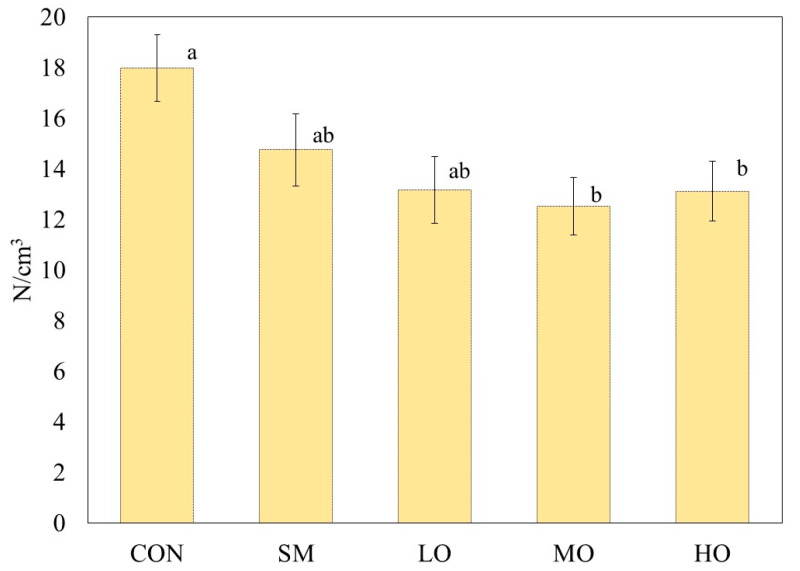
Means (± S.E.) of compression strength (N/cm^3^) in m. *Longissimus lumborum* of male lambs unsupplemented (CON) or supplemented with monensin (SM), or three different levels of oregano essential oils (LO = 0.2 g/kg of DM; MO = 0.3 g/kg of DM or HO = 0.4 g/kg of DM). Compression strength analyses were performed after 7 d of simulated retail display in modified atmosphere packaging (O_2:_CO_2_, 75:25%). ^a, b^ Different superscripts mean significant difference (*p* ˂ 0.05) among treatments within variable.

OEO dietary supplementation reduced the compression force (Figure 2) of lamb (*p* = 0.03). The meat with the medium level of OEO (MO) and the control (CON) meat had the lowest and highest compression strength values, 12.51 ± 1.13 and 17.98 ± 1.32 N/cm^3^, respectively. At day 7, the meat with a high level of OEO (HO) presented the lowest compression strength and the highest oxidation rate. Therefore, a greater PUFA deposition (*p* < 0.05) could be assumed.

### 2.3. Colour

**Figure 3:**
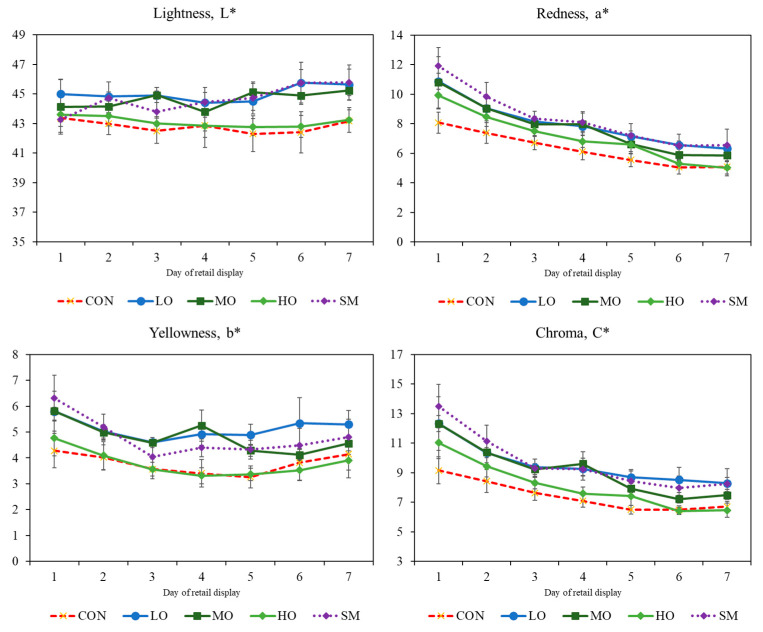
Colour coordinates (L* = lightness, a* = redness, b* = yellowness, and C* = Chroma. ± S.E.) in m. *Longissimus lumborum* of male lambs unsupplemented (CON) or supplemented with three different levels of oregano essential oils (LO = 0.2 g/kg of DM; MO = 0.3 g/kg of DM; or HO = 0.4 g/kg of DM) or monensin (SM), during 7 d of simulated retail display in modified atmosphere packaging (O2:CO2, 75:25%).

The interaction between treatment and day of shelf life was not significant (*p* > 0.05). Treatment and storage time affected the colour of *L. lumborum* (*p* < 0.001). Seven d of storage in 75% O_2_ packs negatively affected (*p* < 0.05) a *, b * and c *, decreasing the values of the three colour coordinates (Figure 3).

The value of a* differed among treatments (*p* < 0.001). SM, MO, and the meat with a low level of OEO (LO) had the highest values during storages (Figure 3, *p* ˂ 0.001). SM, MO, and LO had also the higher b* values throughout storage compared to CON and HO (Figure 3). Treatment also affected C*. In this case, SM, MO, and LO were the highest (*p* < 0.001); meanwhile, CON and HO were similar (*p* = 0.03).

**Figure 4:**
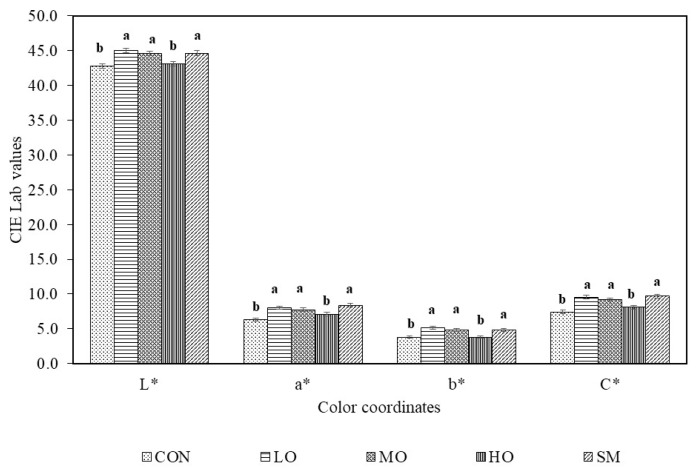
Means (± S.E.) of colour coordinates (L*, lightness; a*, redness; b*, yellowness; C*, saturation) in m. *Longissimus lumborum* of male lambs unsupplemented (CON) or supplemented with three different levels of oregano essential oils (LO = 0.2 g/kg of DM; MO = 0.3 g/kg of DM; or HO = 0.4 g/kg of DM) or monensin (SM), with 7 d of simulated retail display in modified atmosphere packaging (O_2_:CO_2_, 75:25%). ^a, b^ Different superscripts mean significant difference (*p* ˂ 0.05) among treatments.

These statements can be confirmed with the means of the colour coordinates by treatment (Figure 4). It can be established that the dietary addition of 0.2–0.3 g/kg dry matter (DM) OEO and SM (commercial dosage) reduces colour loss after 7 d under MAP.

### 2.4. Fatty Acid Profile

In general, the inclusion of OEO or SM affected the fatty acid profile (*p* > 0.05). Eighteen FAs were analyzed, and their percentage was calculated relative to all FAs (Table 1). Differences were observed (*p* < 0.05) among treatments in a single FA. The treatment modified the percentage of C16:1n7, C20:1n9, and C22:6n3. Furthermore, it also modified the content (mg/kg of muscle) of C14:0, C16:0, C18:0, C16:1n7, C18:1n9c, C18:1n6c, C20:1n9, and C18:2n6c (Table 2).

HO decreased the concentration of C14, C16, and C18 (*p* < 0.05); meanwhile, MO increased (*p* < 0.05) them. LO and SM did not alter the concentrations of these acids when compared to CON (*p* > 0.05, Table 2). Higher concentrations of C14, C16, C18, C16:1n7, C18:1n, C20:1n9, and C18:2n6t in the total fat of *Longissimus lumborum* were observed (*p* < 0.05) in MO meat compared to the control group.

Concentrations (Table 2) of monounsaturated fatty acids (MUFAs); C16:1n7, C18:1n9c and C20:1n9 were different among treatments (*p* < 0.05). MO increased the concentration of these MUFAs (*p* < 0.05), while HO decreased the concentrations (*p* < 0.05). HO, LO, and CON had similar low levels, whereas SM and MO had higher values (*p* < 0.05).

The treatment (*p* < 0.05) affected the concentration of linoleic acid (C18:2n6t) in total fat. MO increased concentrations of C18:2n6t (*p* < 0.05, Table 2). The lowest concentrations of C18:2 were in HO meat.

## 3. Discussion

### 3.1. Lipid Oxidation

In the present study, the incorporation of OEO at levels of 0.2 and 0.3 g/kg DM or monensin in the lamb diet had a similar effect on the lipid oxidation of lamb. The control treatment was slightly higher in malonaldehyde formation in meat. However, this difference was not statistically different from the control treatment. Notwithstanding, the result is still promising as far as the application of OEO concerns, since replacing OEO for monensin in lamb diets shows to be a beneficial choice.

However, meat from male lambs fed with a high level of OEO had significantly higher malonaldehyde (MDA) formation when compared to the control treatment.

The effectiveness of essential oil in preventing oxidation in lamb meat has also been reported by Nieto et al. [33]. They tested distilled dietary rosemary leaf (DRL, 0%, 10% and 20%) to prevent lipid oxidation and the sensory deterioration of cooked lamb, under retail display conditions. Cooked lamb fillets were stored at 0, 2, or 4 d (4 °C) in a display cabinet and then reheated, simulating catering practices. The cooked lamb suffered rapid lipid oxidation and odour and flavour spoilage associated with slight rancidity and warmed-over flavour. DRL feeding delayed lipid oxidation (thiobarbituric acid reactive substances, or TBARS) and volatile compounds more effectively in the first two d of storage. Percentages of 10% and 20% of DRL provided equal antioxidant capacity.

These positive effects of essential oil have also been found in bovine meat. Rivaroli et al. [34] fed crossbred young bulls with different doses of an essential oil blend (oregano, garlic, lemon, rosemary, thyme, eucalyptus, and sweet orange). They found that a dose of 3.5 g/animal/d decreases lipid oxidation. However, higher doses could have a pro-oxidant effect, and they are not recommended in feedlot animals.

Antioxidants that interact with reactive oxidant species (ROS) might become pro-oxidants, causing lipid and protein oxidation [35,36]. Low concentrations of essential oils might prevent this, and antioxidant activity is kept as observed in the present study, where low and medium oregano oil doses (0.2 and 0.3 g/kg DM diet) resulted in lower TBARS values, and the high doses produce an increase of lipid oxidation.

It is important to mention that in the present study, the lipid oxidation is considered still low (TBARS values lower than 2.0), which is in agreement with the report of Campo et al. [37]. They revealed that the TBARS value of 2.0 (2 mg MDA/kg meat) could be considered the threshold where the rancid flavour overpowers beef flavour. Therefore, it is considered as the maximum level for the positive sensory perception of beef. These authors indicated that from that point onwards, it can expected for beef to be rejected due to a strong sensory perception of lipid oxidation.

In physiological conditions, mammals constantly produce reactive oxygen species (ROS). Low concentrations of ROS are essential for several physiological processes, including protein phosphorylation, apoptosis, and cellular defence against microorganisms [38]. Oxidative stress refers to a lack of balance between the production of ROS and the level of antioxidants. Domestic animals are frequently exposed to oxidative stress, especially under intensive breeding systems [39]. Oxidative stress is responsible for numerous disease processes in animals. Many secondary metabolites formed by plants serve as defence agents against physiological and environmental stressors, and pathogenic microorganisms [40]. The main molecules responsible for the antioxidative properties of herbs and spices are phenolic substances. In particular, *Origanum vulgare* is an herb rich in phenolics [41].

Essential oils are rich sources of natural antioxidants, such as the phenolic compounds, and due to their high redox properties and chemical structure, they affect lipid metabolism in animal tissues by exerting beneficial effects on the antioxidant enzyme activity. Furthermore, phenolic compounds also prevent the production of reactive oxygen species and the off-flavors that are formed from the oxidation of polyunsaturated fatty acids [42]. Dietary supplementation with EOs is a simple and convenient strategy to uniformly introduce natural antioxidants into phospholipid membranes, where they may effectively inhibit the oxidative reactions by preventing the formation of radicals, and it appears to be a more effective way of slowing down hte lipid oxidation of animal products compared to post-mortem addition [43,44,45].

Other benefits of OEO have been stated in the literature. OEO modifies ruminal microflora, which also modifies the concentration of ruminal volatile fatty acid. Fat deposition (mainly unsaturated fatty acids, UFAs) is promoted when the concentration of propionic acid decreases and the acetic acid increases. Under some circumstances, UFAs are more susceptible to oxidation [46,47,48], and they may promote the formation of MDA in absence of antioxidants, as observed in this study (Figure 1).

As it has been previously pointed out, the structure of some lipid components from the essential oils changes as they transit through the digestive tract, and if they are absorbed in the intestine, the lipid profile and the oxidative stability of the meat might be modified [46,47,48,49]. In the present study, monensin has a similar effect to that of OEO in terms of lipid oxidation. This indicates that OEO could safely replace monensin in lamb diets, with the advantage of being a natural additive that promotes other positive changes in lamb, such as colour and shelf life preservation. OEO supplementation demonstrated lipid antioxidant activity in fresh lamb meat. OEO improves the antioxidant activity, which has an influence on retarding the lipid meat oxidation during refrigerated and long-term frozen storage. This process could be explained by carvacrol and thymol action on the permeability of cell membranes, and by the transformation of lipid and hydroxyl radicals into stable products [29]. This effect was supported in the present study.

The antioxidant effect of dietary OEO supplementation has also been demonstrated in poultry [44,50,51]. Moreover, OEO has been studied as an ingredient in meat formulations. In lamb burgers, the addition of 24 mL/kg of oregano extract is recommended as a natural antioxidant in replacement of sodium erythorbate, and the product has good acceptability [52].

### 3.2. Compression Strength

The tenderness of meat has been associated with intramuscular fat (IMF) content [53], and the increase of monounsaturated fatty acids (MUFAs) and PUFAs concentration in IMF could reduce the compression force of meat, thus producing more tender meat and, in this way, improving the quality.

Some of the intrinsec main factors that influence meat texture are the content and solubility of collagen, sarcomere diameter, intramuscular fat content, and proteolysis by calpains during ageing, among others [54]. The dietary inclusion of OEO decreases the concentration of acetic acid and increases propionic acid in rumen, which favours fat deposition [55] and improves meat tenderness. An increased quantity of subcutaneous fat and intramuscular fat decreases the rate of temperature decline, enhances the activity of autolytic enzymes in the muscle, lessens the myofibrillar shortening, and thereby increases the tenderness of cooked meat [56]. In the present study, it can be assumed that differences in tenderness between CON and MO are related to intramuscular fat deposition, since MUFA and PUFA are oilier in texture than saturated fatty acids. Apparently, the MO inclusion promoted a greater amount of MUFA and PUFA in the meat.

There are no other studies showing an improvement of lamb tenderness when animals were fed OEO. In the study of Simitzis et al. [57], the dietary oregano essential oil supplementation on lamb did not influence the tenderness of *Longissimus thoracis* muscle. Demirel et al. [58] reported that the effect of oregano oil was not significant on carcass and lamb meat quality attributes.

Contrasting effects of OEO on the tenderness and shear force of meat from other species are reported. Cheng et al. [59] observed that dietary OEO enhanced the tenderness and overall acceptance of pork. Forte et al. [60] showed that dietary oregano essential oil increased the meat tenderness, but it did not modify other pork quality traits, such as the pH, colour, drip loss, and cooking loss. However, OEO improved consumer perceptions of the meat quality, such as consistency and overall liking. In contrast, Ranucci et al. [61] evaluated a plant extract mix (chestnut and oregano essential oil) in a pig diet and evaluated the pig performance and meat quality. The fresh meat colour, pH, and WB shear force was not affected by OEO supplementation. Simitzis et al. [29] did not find any change in the meat shear force and sensory traits of meat from pigs supplemented OEO. As well, Rossi et al. [62] reported an enhancement of sensory attributes in meat from pigs supplemented plant extract (*Lippia* spp.) but did not find any tenderness improvement in the meat.

When adding essential oils to meat products, it has been pointed out that protein oxidation reduces meat tenderness, but the essential oils of oregano and rosemary can protect the thiols in pork patties and reduce the disulphide crosslinks of the myosin heavy chains, avoiding the tenderness reduction of meat [63].

Finally, Lei et al. [64] demonstrated that the addition of essential oil-cobalt had a significant effect on the meat quality of tested goats. Similarly, Velasco et al. [65] found that the incorporation of dietary dry oregano at 1% and 5% in the diet of Boer goats did not affect the meat quality characteristics.

### 3.3. Colour

The addition of the OEO and/or monensin in the lamb diet influences the colour (L*, a*, b*, and C*) parameters of the meat. According to recent reports by Payne et al. [66], the colour values in finishing lambs (240 d old) are L* = 34.3, a* = 5.7 and b* = 16.9. The L* values in the present study are higher (L* = 40), meaning a lighter meat. The high L* value could be attractive to consumers that prefer lighter meat [34]. A positive result could be that yellowness (b*) was relatively lower compared to Payne et al. [66], since consumers do not expect to find high b* in fresh meat. Lightness (L*) was higher in the meat from oregano and monensin treatments compared to the control. As noted by Rivaroli et al. [34], in feedlot-finished young bulls that were fed with essential oils, L* values were superior to the other literature data of cattle finished in feedlot.

Colour is one of the most important quality characteristics to determine the consumer decision for purchasing meat. The natural colour of meat is produced by the myoglobin and hemoglobin pigments. These three components that define the colour of meat are all highly susceptible to oxidation [67,68]. An unattractive brown colour can result from the oxidation of red oxymyoglobin to metmyoglobin. The mechanisms that modify pigment distribution in animal tissues could be activated by lowering hemoglobin oxidation by dietary OEO supplementation [57]. Antioxidants have the ability to retard meat colour deterioration by extending the red colour and delaying metmyoblobin formation. Simitzis et al. [57] included 1 mL OEO/kg in lambs diet and found higher a* and b* values. In lamb meat, Nieto et al. [33] indicates that lambs fed with 3.7% and 7.5% of oregano leaves produced significant differences regarding the colour values. In this study, as the storage period was prolonged, the L* and b* values increased and the a* value decreased. Similarly, Simitzis et al. [29] pointed out that supplementing lamb diets with OEO resulted in significant effects on meat colour (L*, a*, and b*).

Different results have been found in other animal species. The colour of pork patties was investigated by Carpenter et al. [69]. They did not find significant changes in colour parameters by the addition of grape seed and bearberry extracts to the diet. Similar results were obtained for fresh chicken breast meat [25], whereas the incorporation of rosemary and oregano extracts in pig rations resulted in significant differences in the luminosity of meat.

Similar results have been reported by Camo et al. [24], who reported that the packaging of lamb meat using rosemary and oregano extracts resulted in the difference in meat redness of the treated animals compared to the controls. Intrinsic characteristics of the animals have also an effect on meat colour. Lamb meat colour changes by body weight, sex, and breed [70]. In this way, Hopkins and Fogatry [71] found that the colour of the m. *Longissimus thoracis* varied with breed. Based on the findings obtained in this study, the effect of OEO on meat colour parameters was found to be in agreement with the literature and within the reference ranges.

Possibly, components from OEO accumulate on the meat, as it has been reported in non-ruminants [72]. Essential oils have an antioxidant activity when used directly on the meat or supplemented ante-mortem [46,73,74], which may protect meat pigment from oxidation throughout storage. If dietary OEO are accumulated in meat, it might mean that they passed the rumen without being degraded. Alternatively, colour might remain stable, as carvacrol supports the activity of glutathione peroxidase and superoxide dismutase, which are two of the most important antioxidant enzymatic complexes in mammals [75].

It is important to highlight that dietary MO not only maintained a higher and more stable redness, yellowness, and saturation during storage, but it also reduced the compression force, and, although not significant (*p* > 0.05), lower TBARS were observed. Improvement of the oxidative stability of MO meat was shown by the stable colour during storage. Dietary antioxidants such as tocopherol deposited in meat may avoid rancidity or the oxidation of tissue components [76]. Carvacrol has a high antioxidant activity [65]. It is possible that the antioxidant activity of OEO is more related to protein protection (pigments) than to lipid components. Some spices and their extracts such us oregano have a high antioxidant activity due to their phenolic compound content, which improves the nutritive value and the quality of meat, because they prevent meat oxidation [65].

Moura et al. [77] evaluated dietary monensin (SM) and incrementing levels of copaiba (*Copaifera* spp.) essential oil (CO) on nutrient intake, time spent eating and ruminating, performance, carcass traits, and the meat quality of feedlot lambs. They observed that the addition of CO at 1.5 g/kg increased Warner Bratzler shear force and decreased L* intensity in *Semimembranosus* meat in comparison to SM.

### 3.4. Fatty Acid Profile

The supplementation treatments, SM and MO, modified the fatty acid profile compared to the other treatments, whereas HO treatment modified the fatty acid profile undesirably. The similarity between SM and MO might imply that as they modify the rumen environment, the growth rate of rumen microflora changes, resulting in changes in the fermentation profile [49,55,78]. These changes impact the fatty acid profile [79], as it has been reported that monensin was at least partially effective to inhibit the biohydrogenation of unsaturated FAs in the rumen. This consequently increased the percentage of n-6 and n-3 PUFAs and conjugated linoleic acid in milk.

SM (Rumensin^®^) in ruminant diets [55] and essential oils have bactericidal or bacteriostatic effects [13]. The antibacterial effect is more evident in Gram-positive bacteria, where the cell membrane acts directly with hydrophobic components [80]. SM and some compounds in essential oils are lipophilic; hence, they do not penetrate the membrane of Gram-negative bacteria [81,82]. However, Gram-negative bacteria are not completely resistant to the lipophilic compounds in essential oils, because low molecular weight molecules can interact with the cellular lipid bilayer [82]. Thymol and carvacrol can also disintegrate the external membrane of Gram-negative bacteria [83]. Hence, SM and essential oils affect equally Gram-positive and Gram-negative bacteria, but they use different pathways. The levels of essential oil inclusion are fundamental, because it has been reported that low levels are not enough to modify the ruminal microflora and high levels reduced significantly the bacterial counts, while neither of them change the ruminal fermentation rate [78].

In this regard, several authors have already shown the mechanism of SM inducing ruminal environmental changes. It has been pointed out that SM modifies the ruminal and intestinal microflora, which causes a higher nitrogen and carbon retention in the animal [3]. Additionally, SM promotes the growth of propionic acid-producing microorganisms. Therefore, the concentration of propionic and butyric acids increase, while acetate decreases in ruminal fluid. This leads to an acetate:propionate ratio decline [3,84,85,86], which in turn favours the recovery of energy used by the animal [79]. Additionally, SM reduces the formation of methane and lactic acid produced by other microorganisms [87,88].

In the present study, most of the FAs that were statistically different are saturated or monounsaturated. This might indicate that triglycerides are accumulating in the intramuscular adipocytes within the neutral lipid fraction. Nevertheless, phospholipidic variations may take place, considering that this fraction is easily altered with the diet [89,90]. An advantage of monensin is that it does not only change the microbial populations in the rumen to such levels that the fatty acid profile is modified, but it also changes the digestibility of nutrients and the utilisation of proteins [3]. Ionophores such as SM alter the fat deposition in beef, particularly arachidonic (C20:4) and linolenic (C18:3n3) acids [91,92]. Furthermore, in bovine milk, SM also changes the amount of fat and increases C18:2 [93]. However, an outstanding characteristic of OEO is that its active components (carvacrol and thymol) of OEO are potent antimicrobials affecting populations such as *E. coli*, *Staphylococcus aureus*, *Salmonella typhimurium*, protozoa, fungi, *Ruminococcus fibrisolvens* and *Fibrobacter succinogenes*, which modifies ruminal fermentation and is fundamental in the conversion of dietary nutrients to muscle tissue [11,12,13]. Specifically in sheep, there is evidence that carvacrol decreases acetate concentrations and increases propionate and butyrate. Both are volatile fatty acid precursors of muscle and fat components in the animal [14].

Other essential oils have also been studied in lamb nutrition, and their results are promising. Parvar et al. [94] investigated the effects of *Ferulago angulata* (chavil) essential oil (FAE) dietary supplementation on growth performance, meat quality characteristics, and the fatty acid composition of *longissimus* muscle (LM) in fattening lambs. They found that the supplements increased the concentrations of PUFA and decreased SFA contents in meat. Lambs that used diets containing FAE had a lower n-6:n-3 fatty acid ratio compared to the control treatment. They concluded that FAE (up to 750 mL/kg DM) can be used in diets without adverse effects on physical parameters or the chemical composition of meat, and it enhanced the anti-oxidative status of lamb’s meat. On the other hand, negative effects of monensin in sheep have been observed. A study of lamb supplementation with monensin (zilpaterol hydrochloride, ZH; 0 or 10 mg/lamb daily) showed a decrease in the content of C20:5n3 (eicosapentaenoic acid), C22:6n-3 (docosahexaenoic acid), and total omega-3 fatty acids, compared with the zero ZH group [95].

In monogastric animals such as chickens, the inclusion of carvacrol and thymol fat increases PUFA and decreases SFA in breasts [75]. In this study, the PUFAS concentration was not different (*p* > 0.05) between the control and monensin treatment. However, the PUFAs concentration was higher in MO. Promising results of OEO have also been reported in pork. Cheng et al. [59] reported that dietary OEO enhanced the sensory attributes and anti-oxidative status of pork meat by improving IMF and n-3 PUFA proportion and antioxidant capacity.

## 4. Materials and Methods

### 4.1. Animal Handling and Treatment Description

Twenty male lambs (Dorper x Pelibuey. Initial body weight, 26.2 ± 3.9 kg) were randomly assigned to one of five treatments (*n* = 4 per treatment); CON: control, basal diet; SM: basal diet + 33 mg/kg monensin sodium (Rumensin 200^®^); LO: basal diet + 0.2 g/kg DM (dry matter) of OEO; MO: basal diet + 0.3 g/kg DM of OEO, and HO: basal diet + 0.4 g/kg DM of OEO. The basal diet was formulated for 27 kg-lambs to gain 250 g daily [96], and it consisted of 20% alfalfa hay and 80% concentrate DM basis (Table 3). OEO (62.7% carvacrol concentration) was extracted from the leaves (*Lippia* S. *berlandieri*) by steam distillation and obtained from Natural Solution^TM^ Jimenez, México. The diet adaptation period lasted 15 d, and the experimental period was 70 d.

Animals were housed in individual pens (equipped with feeding and drinking troughs) and fed twice daily (8:00 and 16:00 h). All experimental procedures with the animals complied with the institutional Bioethics code and Animal Welfare Guidelines, fulfilling the Official Mexican Norms. The protocol was approved by the Institutional Bioethics and Animal Welfare Committee on September 21, 2007, with official number P/302/2017. Description and declarations in this document also followed the Animal Research: Reporting of In Vivo Experiments (ARRIVE) guidelines.

At day 71 (average body weight 45.9 ± 2.82 kg) and after a 12-h fasting period, the animals were electrically stunned and slaughtered by exsanguination using conventional methods in the Meat Science Complex, Department of Animal Science and Ecology (UACH). The dressed carcasses were hung by Achilles tendon suspension in a chiller at 2 °C. At 48 h post-mortem, a portion of *Longissimus lumborum* (LL, 15 cm approx.) was excised longitudinally (after the 12^th^ rib) from the right half of the carcass. The portions of *LL* were vacuum packed (Easy-pack, Rhino, Germany) in 7 µm thickness pouches and fast-frozen (−20 °C) until further analyses.

After two weeks, frozen muscles were sliced transversally to obtain 16 steaks (2 cm thickness) transversal to the muscle fibre direction. Steaks were analysed as follows: the two cranial steaks were tested for lipid oxidation, the next 2 were tested for colour, the following three were tested for texture, and the last 2 were tested for the fatty acid profile.

Samples for lipid oxidation, colour, and texture were thawed at 3 °C for 24 h. Then, to simulate commercial retail display, samples were packed under a modified atmosphere (MAP, 75% O_2_-25% CO_2_, PVC Cryovac^®^ trays, PET-PVDC-PE top lidding. Rhino 4 sealed packer. USA) and placed in a chilled storage (3 °C, artificial led light, 12 h/d, 700 lx). Samples for fatty acids were re-packed in vacuum bags and kept frozen for 7 d more until fatty acid analysis. All measurements were performed in triplicate except for the compression test, for which measurements were taken at least six times.

### 4.2. Lipid Oxidation

Lipid oxidation was determined after 7 d of simulated commercial retail display by TBARS (thiobarbituric acid reactive substances), and the values were expressed as mg malonaldehyde (MDA) per kg of meat, according to the distillation method [97].

### 4.3. Compression Strenght Analysis

To determine the compression strength of the samples, the methodology of the American Meat Science Association Guidelines [98] was followed. MAP chilled stored samples were placed in sealed plastic bags and cooked in a water bath (Fisher Scientific^®^ mod. Isotemp 215, Waltham, MA, USA) until an internal temperature of 72 ± 1 °C was reached at the geometric centre. Temperature was monitored with a thermocouple wire, which was attached to an infrared digital thermometer (Fisherbrand™ Traceable™ Infrared Thermometer with Trigger Grip). Subsequently, cooked steaks (2 cm thickness) were stored at 1 °C for 24 h. Compression strengt was determined by the “punch and die” method [99], which was modified by Jones et al. [100]. Steaks (2 cm thickness) were perforated transversally (parallel to muscle fibers) at least 6 times, and compression values were averaged per every steak (3 steaks per animal). Compression strength analysis was performed with a TA.XT2*i* texture analyser (Stable Micro Systems, Surrey, UK), attached to a 30 kg load cell, and set with a 20 mm cylindrical probe (Crosshead speed of 100 mm min^−1^ at 3 cm of distance). Data are expressed in Newtons/cm^3^.

### 4.4. Colour

Colour (L *, a *, b *, and c *) was evaluated daily for 7 d. Measurements were taken thrice in MAP raw meat with a colorimeter (Minolta^®^ Konica Minolta Camera, Tokyo, Japan), 8 mm Illuminant C. Standard observer, C: Y = 94.2, x = 0.3130 and y = 0.3190following the methodology of the Commission Internationale l’Eclairage with the CIELAB scale [101].

### 4.5. Fatty Acids (FA) Profile Analysis

Fatty acid profile analysis was carried out in the steaks without simulated retail display. Lipid extraction was carried out following Bligh and Dyer [102,103]. FA derivatization was obtained by saponification, methylation, and esterification [104]. FA were analysed by gas chromatography (Claurus 400 Perkin Elmer, Waltham, MA, USA), with a polar column (100 m × 0.25 mm × 0.20 μm; Sigma, Bellfonte, PA, USA). Peak identification was achieved by comparing the retention times of the unknowns with the standard SupelcoTM 37 Component FAME mix (Sigma).

### 4.6. Analysis of Data

Variables measured only once (TBARs values, compression values, fatty acid, and concentrations) were analysed with a one-way ANOVA, where diet (5 treatments) was the independent variable. If the treatments were significantly different (*p* < 0.05), means were compared with a Tukey test. The effect of diet on colour (L *, a *, b*, an c *) was analysed with a general linear mixed model, considering the day of the storage as random variable and diet (5 treatments) as a fixed effect. All analyses were performed using the ‘R’ statistical software version 3.6.0 [105].

## 5. Conclusions

This study demonstrated that the dietary inclusion of 0.3 g (per kg of DM) of OEO increases lamb tenderness and, similarly to monensin and low oregano oil (0.1 g/kg DM) supplementation, it preserves the meat colour after 7 d of storage. Thus, oregano oil could be an alternative to monensin in lamb diets. The supplementation of oregano essential oil also significantly affected the fatty acid profile, increasing the content of C16:1n7, C18:1n9c, C20:1n9, and C18:2n6 in meat. This study demonstrated that oregano essential oil was beneficial for lamb meat quality. Overall, this study confirmed the remarkable beneficial effect of oregano essential oil on the colour, tenderness, lipid oxidation, and fatty acid profile of lamb meat, which is of significant importance, aiming to evaluate the benefits of phytochemicals to replace monensin and obtaining advantages in lamb meat quality. Further research is needed to identify the main metabolic pathway of oregano essential oil as well as the crucial active components that favourably alter the quality characteristics in lamb meat.

## Figures and Tables

**Table 1:** Percentage of fatty acids in the total fat of *Longissimus lumborum* of male lambs unsupplemented (CON), supplemented with three different levels (HO = 0.4 g/kg DM; MO = 0.3 g/kg DM; LO = 0.2 g/kg DM) of oregano essential oils or supplemented with monensin (SM) at commercial doses.

Fatty Acid	Treatments					
	CON	SM	HO	MO	LO	*p*-Value
C14:0	1.95	1.68	1.99	2.00	1.78	0.60
C14:1	0.11	0.10	0.09	0.10	0.10	0.99
C16:0	25.00	23.69	23.26	25.64	25.07	0.49
C16:1n7	1.00^ab^	1.35^a^	0.87^b^	0.91^ab^	1.06^ab^	0.04
C18:0	14.69	15.07	15.04	15.87	14.90	0.94
C18:1n9t	2.31	2.22	1.63	2.84	2.20	0.19
C18:1n9c	44.17	46.28	45.83	44.89	46.24	0.52
C18:2n6t	0.25	0.24	0.24	0.22	0.21	0.97
C18:2n6c	3.10	3.32	3.28	2.69	2.85	0.60
C18:3n^6^	0.09	0.06	0.07	0.05	0.03	0.78
C18:3n^3^	0.06	0.04	0.04	0.04	0.04	0.17
C20:0	0.38	0.27	0.18	0.25	0.32	0.10
C20:1n^9^	0.72^ab^	0.62^b^	0.69^ab^	0.82^a^	0.58^b^	0.01
C20:3n^3^	0.05	0.05	0.08	0.04	0.04	0.35
C20:4n^6^	0.14	0.13	0.08	0.05	0.08	0.46
C20:5n3	1.68	1.48	1.81	1.25	1.12	0.13
C22:5n^3^	0.86	0.39	0.53	0.33	0.46	0.27
C22:6n3	0.30^ab^	0.23^ab^	0.86^a^	0.21^b^	0.23^ab^	0.03

^a,b^ Different superscripts mean significant difference (*p* ˂ 0.05) among treatments (columns).

**Table 2:** Concentration of fatty acids (mg/kg of muscle) in total fat of *Longissimus lumborum* of male lambs affected by supplementation of three different levels (HO = 0.4 g/kg DM; MO = 0.3 g/kg DM; LO = 0.2 g/kg DM) of oregano essential oils or monensin (SM) at commercial doses *vs.* a control diet (CON).

Fatty Acid	Treatments					
	CON	SM	HO	MO	LO	*p*-Value
Saturated						
C14:0	13.99^b^	14.47^ab^	9.67^b^	23.50^a^	13.18^b^	0.042
C16:0	172.7^b^	200.1^ab^	120.88^b^	289.77^a^	183.74^ab^	0.031
C18:0	94.93^b^	121.7^ab^	71.94^b^	164.17^a^	103.58^ab^	0.043
Mono-unsaturated						
C16:1n7	6.60^ab^	10.68^a^	4.31^b^	9.51^a^	7.14^ab^	0.024
C18:1n9c	287.41^b^	369.6^ab^	221.58^b^	472.97^a^	321.59^ab^	0.043
C18:1n9t	14.67	16.82	14.39	8.12	30.19	0.007
C20:1n9	2.90^b^	3.54^b^	4.21^b^	7.88^a^	4.46^b^	0.001
PUFA’s						
C18:2n6t	18.74^ab^	24.88^ab^	14.04^b^	27.10^a^	18.66^ab^	0.043

^a,b^ Different superscripts mean significant difference (*p* ˂ 0.05) among treatments (column) in the same fatty acid.

**Table 3:** Ingredients and chemical composition (DM basis) of diets of finishing hair lambs supplemented with Carvacrol.

	Treatment				
Ingredients (%)	Control	Monensin	Low Oil	Medium Oil	High Oil
Rolled sorghum	36.32	36.32	36.32	36.32	36.32
Soybean meal	34.79	34.79	34.79	34.79	34.79
Alfalfa hay, full bloom	20	20	20	20	20
Molasses cane	5	5	5	5	5
Corn gluten (60% CP)	2	2	2	2	2
Calcium carbonate	0.883	0.883	0.883	0.883	0.883
Salt	0.5	0.5	0.5	0.5	0.5
Mineral premix 1	0.5	0.5	0.5	0.5	0.5
Essential oil (Carvacrol, g/Kg MS)	0	0	0.2	0.3	0.4
Monensin (pmm)	0	33	0	0	0
Calculated chemical composition (% DM basis)					
CP	23.68	23.68	23.68	23.68	23.68
ME (Mcal/kg)	2.739	2.739	2.739	2.739	2.739
Ca	0.791	0.791	0.791	0.791	0.791
P	0.453	0.453	0.453	0.453	0.453

CON: 0 g/Kg MS Carvacrol; M: 33 ppm/Kg MS Monensin; Low: 0.2 g/Kg MS Carvacrol; Medium: 0.3 g/Kg MS Carvacrol; High: 0.4 g/Kg MS Carvacrol. ^1^ P 12%; Ca 11.5%; Mg 0.6%; Mn 2160 ppm; Zn 2850 ppm; Fe 580 ppm; Cu 1100 ppm; I 102 ppm; Co 13 ppm; Se 9 ppm; Vitamins: A 22,000 UI/Kg; E 24,500 UI/Kg.
